# The effectiveness of joint crisis plans for people with borderline personality disorder: protocol for an exploratory randomised controlled trial

**DOI:** 10.1186/1745-6215-11-18

**Published:** 2010-02-23

**Authors:** Paul Moran, Rohan Borschmann, Clare Flach, Barbara Barrett, Sarah Byford, Joanna Hogg, Morven Leese, Kim Sutherby, Claire Henderson, Diana Rose, Mike Slade, George Szmukler, Graham Thornicroft

**Affiliations:** 1 Institute of Psychiatry, King's College London, Sir David Goldberg Building, De Crespigny Park, SE5 8AF, London, UK

## Abstract

**Background:**

Borderline Personality Disorder (BPD) is a common mental disorder associated with raised mortality, morbidity and substantial economic costs. Although complex psychological interventions have been shown to be useful in the treatment of BPD, such treatments are expensive to deliver and therefore have limited availability and questionable cost-effectiveness. Less complex interventions are required for the management of BPD. A Joint Crisis Plan (JCP) is a record containing a service user's treatment preferences for the management of future crises and is created by the service user with the help of their treating mental health team. These plans have been shown to to be an effective way of reducing compulsory treatment in people with psychosis. However, to date they have not been used with individuals with BPD. This exploratory trial will examine whether use of a JCP is an effective and cost-effective intervention for people with BPD for reducing self-harm.

**Methods/Design:**

In this single blind exploratory randomized controlled trial, a total of 120 participants (age >18 years with a primary diagnosis of DSM-IV borderline personality disorder) will be recruited from community mental health teams and, after completing a baseline assessment, will be assigned to one of two conditions: (1) a Joint Crisis Plan, or (2) treatment as usual. Those allocated to the JCP condition will take part in a facilitated meeting, the purpose of which will be to agree the contents of the plan. Following the meeting, a typed version of the JCP will be sent to the patient and to any other individuals specified by the participant. All participants will be followed-up at 6 months. The primary outcome measures are: any self-harm event, time to first episode of self-harm and number of self-harm events over the follow-up period. Secondary outcome measures are length of time from contemplation to act of self-harm, help-seeking behaviour after self-harm, cost, working alliance, engagement with services and perceived coercion. Other outcome variables are quality of life, social impairment and satisfaction with treatment.

**Discussion:**

Results of this trial will help to clarify the potential beneficial effects of JCPs for people with BPD and provide information to design a definitive trial.

**Trial Registration:**

Current Controlled Trials ISRCTN12440268

## Background

Borderline personality disorder (BPD) is characterised by a pervasive pattern of instability in emotional regulation, impulse control, interpersonal relationships, and self-image [1]. The condition occurs globally with a community prevalence of 0.7%, although the prevalence is far higher in clinical populations where it affects up to 20% of psychiatric outpatients [2] and is associated with high rates of co-morbidity and significantly increased health service costs [3,4]. People with BPD are more likely to experience adverse life events and their ability to cope with such events is impaired by poor problem-solving skills [5]. Self-harm is often employed by people with BPD as a way of regulating their level of arousal at times of crises, however, such behaviour puts them at increased risk of suicide [6].

A Joint Crisis Plan (JCP) is a record containing a service user's treatment preferences for the management of future crises. It is developed by a mental health service user in collaboration with staff and aims to increase the service user's level of involvement in their own treatment [7]. Held by the service user, it contains his or her treatment preferences for any future psychiatric crisis, when he or she may be less able to express clear views. The JCP is created at a meeting between the service user, those involved in his or her care, and an independent facilitator not involved in the service user's care. The service user is provided with a template (a list of subheadings the service user may or may not wish to include in the plan) and the team then openly discusses with the service user the advantages and disadvantages of the various subheadings included. The JCP is therefore produced collaboratively between the service user and his or her treating team with the aim of the plan being consulted and followed during a future crisis. JCPs improve the information available to clinical staff about the management of a crisis and empower service users by ensuring that they are actively involved in the generation of their own crisis plan. JCPs have been shown to be an effective way of reducing compulsory treatment in people with psychosis [8], help service users feel more in control of their mental health problems [9] and may also reduce admission rates to hospital [10].

The treatment of people with BPD is frequently characterized by conflicts with health professionals and such conflicts are particularly likely to arise in the management of self-harming behaviour [11]. Moreover, the cessation of self-harming behavious is often associated with an individual's ability to assert greater control over their life [12]. Given that the JCP is designed to facilitate a more collaborative relationship with staff, we hypothesise that JCPs may be an effective way of managing self-harm in people with BPD. In this article, we describe the proposed methods of an exploratory randomized controlled trial (RCT) that is designed to estimate the effectiveness and cost-effectiveness of JCPs on self-harming behaviour in people with BPD and to determine the most effective methods of data collection to inform future studies.

## Methods/Design

### Overview

The "Joint crisis plans for people who **S**elf-**H**arm" (JOSHUA) study is a single centre exploratory randomized controlled trial of JCPs compared with a treatment as usual control condition for people with BPD and a history of self-harming behaviour. The total duration of the study will be two years, to allow for the recruitment to target numbers of participants, provision of the intervention, follow-up assessments, and data analysis, using intention-to-treat methods.

### Aims

The JOSHUA study has the following aims:

1. To determine whether JCPs for people with BPD have a beneficial effect on self-harming behaviour, and to estimate the likely range of effects consistent with the use of JCPs.

2. To determine the cost-effectiveness of JCPs for people with BPD.

### Eligibility criteria

#### Inclusion criteria

i) Age greater than or equal to 18 years

ii) Current contact with a Community Mental Health Team (CMHT)

iii) A primary diagnosis of DSM-IV Borderline Personality Disorder

iv) An episode of self-harm in the previous year

#### Exclusion criteria

(i) Age less than 18 years

(ii) Unable to give informed consent.

(iii) Unable to speak English. Fluency in English is necessary to complete the assessment instruments (many of which have not been validated in non-English languages) and to fully participate in the development of the Joint Crisis Plans.

(iv) Primary diagnosis of psychosis

(v) Current in-patients will not be recruited to avoid any perceived potential coercion to participate, nor any patient subject to a compulsory community treatment order.

No other exclusions will be made, to maximise the external validity of the trial.

### Recruitment and baseline procedures

**Figure 1:**
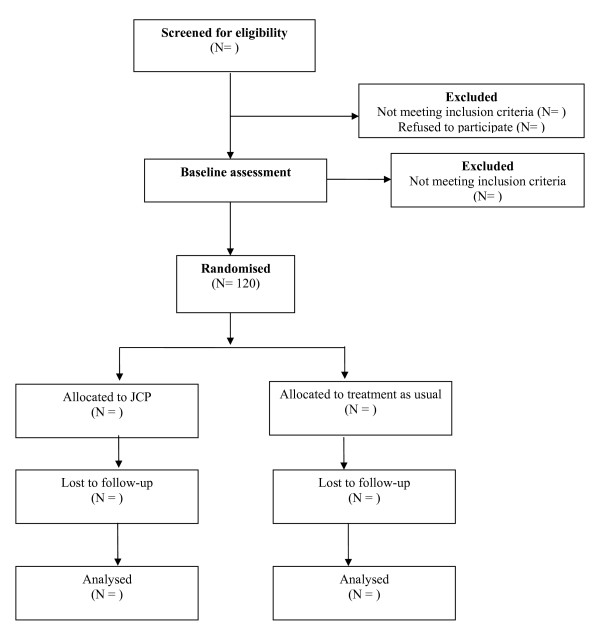
**CONSORT flow chart of JOSHUA trail design**.

The research team will approach local Community Mental Health Teams (CMHTs) and present the study to staff, allowing them to ask questions and become familiar with the study. Team managers will then be asked to identify all potentially eligible participants under the care of their team. Research workers will then approach potential participants through their care coordinator and invite them to a meeting in order to discuss the study in detail. At this meeting they will be given a full description of the study and provided with a written information sheet. If willing to participate, they will be invited to sign the consent form. A baseline interview will then be conducted by the research worker, either at this point or at a subsequent meeting depending on the wishes of the participant. In a pilot study of 13 participants (described further below) in which feasibility of administering the intervention and collecting data were examined, the average duration of the baseline interview was 45 minutes, with no drop-outs. Once the baseline interview has been conducted, the participant will be randomised independently, to either the intervention or control group. Figure 1 displays the flow of participants through the trial.

### Randomisation procedure and methods to minimise bias

Participants will be randomised using the method of minimisation with a random component stratified by alcohol misuse [as measured by the Alcohol Use Disorders Identification Test (AUDIT) [13]] and depression [as measured by the Hospital Anxiety and Depression Scale (HADS) [14]]. The randomisation will be managed by the Clinical Trials Unit (CTU) at the Institute of Psychiatry. Confirmation of eligibility, consent, and baseline data will be obtained prior to randomisation. Bias in the recruitment and randomisation process will be avoided by having randomisation done centrally by the CTU, and hence concealment maintained from the investigators, and by keeping a log of all service users randomised and including them all in the analysis. The research worker collecting outcome data will be blinded to the allocation. Some outcome data will be obtained from electronic patient records, even for participants lost to follow-up interview. This will include any episodes of self harm, hospital admissions and service use recorded in the electronic records. Bias in the collection of outcome data will also be minimised by the use of standardised objective assessments and by blinding the researchers as far as possible to service user allocation. There will be specific instructions to participants and clinical teams not to disclose the treatment details of any study participant. Every effort will be made to maximise the single blindness of research workers, whose 'best guesses' of treatment arm status will be assessed after the final assessment in order to test whether their blindness was successfully maintained.

### Intervention

#### Development of the experimental intervention

The JCP was originally developed for patients with psychotic illnesses. For the purposes of this study, the JCP template and procedure needed to be altered in order to make it relevant to people with BPD. For example, the original JCP template used constructs such as "relapse" which are less applicable to people with BPD. Adaptation of the JCP was informed by three distinct phases. Firstly, a series of focus groups was convened with mental health professionals and service users during which necessary adaptations were discussed. After the JCP template was amended on the basis of the feedback from the focus groups, the amended version was emailed out to members of the focus groups for further refining. Secondly, an email consultation exercise was conducted with leading UK clinical academics (n = 16) to assess the relevance of each item in the JCP menu. The final JCP template contained information for the service user, information for health professionals and details of practical help which the service user might require when in a future crisis. The procedure for developing a JCP for patients with a psychotic illness involved a joint planning meeting with the clinical team, including a doctor, because of the likelihood of treatment preferences or refusals involving medication. However, this was considered to be less relevant to patients with BPD, so the planning meeting would take place with the care coordinator, but a doctor would be consulted if required. Finally, we examined the feasibility of conducting JCP planning meetings and data collection methods with a small convenience sample of BPD service users (n = 13). Results from this case series indicated that not only was the process of developing JCPs feasible, but also that the JCPs were potentially useful - participants in the case series reported using the JCPs when experiencing crises, with some stating that they had reduced their self-harming behaviour since creating their JCP.

#### Administration of the experimental intervention

In the trial, a clinically experienced research worker will organise a meeting with each participant randomised to receive a JCP. The research worker will introduce a list of topics to be considered for inclusion in the JCP and will then organise a meeting between the participant and their care coordinator, when the JCP contents will be finalised. The research worker will then produce a typed version of the JCP and copies will be sent to all those whom the participant specifies. A copy of the JCP will also be attached to each participant's electronic psychiatric case record in order to maximise dissemination of the plan.

#### Control intervention

The participants in the control condition will receive treatment as usual (TAU), as this provides a fair comparison with routine clinical practice and will answer the question of whether JCP use is superior to current standard care. TAU includes, as a part of the Care Programme Approach (CPA), the need for service users to receive written copies of their care plan, including a prescriptive 'crisis contingency plan', in addition to regular contact with a care coordinator or allocated member of the clinical team. We anticipate that the CPA arrangements will be applied equally by routine services to intervention and control groups. Those participants allocated to receive a JCP will also continue to receive usual treatment from their CMHT in addition to creating a JCP. We do not anticipate that TAU will change during the course of the trial and will be documenting what TAU consists of through our collection of service use data.

### Assessments

#### Baseline assessments

(i) Socio-demographic details - age, sex, ethnicity, employment, educational, marital status

(ii) Details of self-harming behaviour over the preceding 6 months - measured using questions from Hawton et al [15]

(iii) Personality disorder status - Structured Clinical Interview for DSM-IV personality disorders (SCID-II) [16] - BPD subsection

(iv) Functional impairment - Work & Social Adjustment Schedule (WSAS) [17]

(v) Engagement with services - Service Engagement Scale [18]

(vi) Working Alliance - Working Alliance Inventory- Short Form (client version) (WAI-S) [19]

(vii) WAI-S (therapist version)

(viii) Health-related quality of life - Euroquol (EQ-5D) [20]

(ix) Satisfaction with care - Client Satisfaction Questionnaire (CSQ) [21]

(x) Perceived coercion - Treatment Experience Scale [22]

(xi) Service use - Adult Service Use Schedule (ADSUS) [23]

(xii) Alcohol use - Alcohol Use Disorders Identification Test (AUDIT) [13]

(xiii) Illicit substance use - Questionnaire on use of illicit substances taken over last year

(xiv) Emotional symptoms - Hospital Anxiety and Depression Scale (HADS) [14]

Additionally, socio-demographic data and length of practice data will be collected for care coordinators, using a proforma developed during the previous pilot work.

#### Follow-up assessments

Six months after randomisation, all participants will be followed-up. A research worker, blinded to treatment allocation, will arrange a convenient time to meet with the participant in order to complete the following measures:

i) Time to first act of self-harm (if any)

ii) Number of self-harm episodes over the preceding 6-month period

iii) Regarding the most recent act of self-harm - length of time from contemplation of self-harm to self-harm act

iv) Regarding the most recent act of self-harm - help-seeking behaviour prior to event (see 10.3)

v) WAI-S (client + therapist)

vi) WSAS

vii) Service Engagement Scale

viii) Treatment Experience Scale

ix) EQ-5D

x) CSQ

xi) ADSUS

### Self-harm follow-up data

Variables (i) and (ii) will be gathered from the following sources:

1. The follow-up interviews with participants at 6-months - participants will be asked to recall the approximate number of self-harm events occurring over the preceding six months. Variables (iii) and (iv) will be gathered using questions derived from Hawton et al [15].

2. Diaries: all participants will be supplied with a simple diary/calendar and will be asked to record all self-harm events on this (by simply circling the days of the month on which self-harm events occurred).

3. Electronic psychiatric case records.

### Sample size

#### Assumptions

For the purposes of the power calculation, the proportion of participants self-harming within the two groups has been chosen as the primary outcome, as this is likely to be the most consistent across the data collection methods, the most complete, and is more amenable to cost-effectiveness analysis. In a previous large RCT (the POPMACT trial) of cognitive therapy versus TAU for people who self-harmed, 36% of patients in the TAU group had a self-harm episode over the first 6 months of the trial [24]. We envisage that the incidence of self-harm in our TAU will be similar to the POPMACT trial. In an RCT of 'green cards' versus TAU for first presentation self-harm, the risk of self-harm after randomisation in the green card group was 37% (95%CI 14% to 97%) of the risk in the TAU group [25]. Given that the green card is not individualised whereas JCPs will be, it will be assumed that the JCP intervention would result in a larger effect, with a lower proportion (one third) of individuals at risk of self-harm since randomisation (33%).

#### Power calculation

Participants will be followed up for a minimum of six months with a predicted 36% and 12% of participants repeating self harm (including serious threats) in the TAU group and JCP intervention group respectively. On the basis of these predictions, an overall sample of 114 (randomised 1:1 to TAU: JCPs) would provide 80% power to detect an observed difference between TAU and JCPs based on a 2-sided test at the 5% significance level. This will be increased to 120 to allow a small loss in the administrative data on self-harm. This sample is also large enough to provide 80% power to detect a constant hazard ratio between the groups of 0.29 with proportions of events in the two groups as stated above, based on the log-rank statistic assuming no accrual rate, a fixed time of follow-up and an estimated 10% dropout.

### Statistical analysis plan

An intention-to-treat analysis will be applied in the first instance (i.e. analysing all available data from randomised participants). The three data collection methods for the outcome of self-harm (i.e., participant interviews, diaries and electronic case records) are likely to differ on the amount of missing data. We will compare proportions of missing data using chi-square tests and agreement between data collection methods using Lin's Concordance Correlation Coefficient. Preliminary effectiveness estimates: Logistic regression will be used to compare proportions of patients that have self-harmed during the follow-up period in the two groups. Analysis of 'time to first self harm' outcome will also be conducted using survival analysis methods; log-rank test for bivariate comparisons and cox's proportional hazards to adjust for gender, stratification variables and time spent on treatment prior to randomisation. Other outcome assessments (continuous and binary) will be compared between the treatment groups at 6-month follow-up using t-tests, chi-square tests or non-parametric equivalents as appropriate, and regression modelling. The primary outcome analysis will be repeated on all three outcome collection methods to determine if conclusions remain the same. We expect little disparity in reporting of 'any self-harm episode' but potentially more in the time of first episode and number of episodes. If conclusions differ by the collection method used we will investigate reasons for the discrepancy. From this analysis we will be more certain of the appropriate collection methods for self-harm data and be better placed to decide on the appropriate outcome measure and estimate effectiveness in a future definitive trial.

### Economic evaluation

The economic evaluation will take a broad perspective, including the cost of all hospital and community health and social care services and contacts with the criminal justice system. Resource use information will be collected using a modified version of the Adult Service Use Schedule (AD-SUS) [23]. These data will also allow us to record what TAU was actually delivered during the trial. Resource use data will be combined with appropriate national unit costs to calculate the total cost of the intervention and control groups. The cost of the JCP will be directly calculated from salaries using a micro-costing approach [26]. Differences in mean total costs between groups will be compared using the standard t-test with ordinary least squares regression used for adjusted analyses and the validity of results confirmed using bootstrapping [27]. The cost-effectiveness of the intervention will be assessed through the calculation of incremental cost effectiveness ratios, using the primary outcome measure and cost-utility will be assessed through the calculation of QALYs based on the EQ-5D. Uncertainty around the cost and effectiveness estimates will be represented using cost-effectiveness acceptability curves [28].

### Process evaluation

A qualitative study will be carried out to explore the processes through which JCPs work in practice. Negotiating JCP content may clarify treatment issues and build consensus between service users and staff. However effects on trust, service user engagement in the process of care including shared decision making, changes to service user self esteem and empowerment, clearer channels of communication between all parties, changes to staff risk perception and/or changes within the culture of the mental health service may also be important. Focus groups will be used to examine people's experience of the JCP and separate service user and health professional focus groups will be convened. We anticipate involving approximately 5-10 service users and 5-10 staff in each of the two groups.

### Ethics

The study protocol was approved by South London REC Office (REC number: 09/H0803/113).

## Discussion

Joint Crisis Plans have been shown to to be an effective way of reducing compulsory treatment in people with psychosis, but to date, no study has examined their effectiveness in people with BPD. Despite the fact that BPD is a prevalent condition associated with increased mortality and substantial economic costs, treatment research in this area is still in its infancy. Complex psychological interventions have been shown to be useful [29], although such treatments are expensive and time-consuming to deliver and this has limited their availability. Less complex interventions are required for the large proportion of BPD people who are not referred to specialist services and JCPs may be an effective and cost-effective intervention [30]. We undertook detailed developmental work with service users, clinicians and academics in order to modify JCPs for people with BPD and to test the feasibility of developing such JCPs in a busy clinical environment. The plans proved to be both feasible and potentially useful and we now plan to test the effectiveness of JCPs in the exploratory trial.

The exploratory trial will gather critical information about key trial parameters necessary to inform the design of a definitive trial, including information on candidate outcome variables, consent and attrition rates and the acceptability of randomising people with BPD. Regarding our choice of primary outcome, currently, there is no gold standard for reliably measuring episodes of self-harm [31]. Accordingly, and to enhance the validity of our data, we will be making use of both contemporaneous and retrospective methods to measure self-harm including self-report, completion of diaries and electronic patient records. It is equally possible that whilst the JCP may not influence self-harming behaviour per se, the production of a JCP might lead to improvements in engagement with services and/or greater satisfaction in the management of future self-harm events. For these reasons, we will also be measuring these outcome domains as secondary outcome variables.

The JOSHUA study has limitations which warrant mentioning. Firstly, it is not a double-blind study design and, as such, the participants and certain members of the research team will be aware of which arm of the trial each participant has been allocated to. However, collection and analysis of follow-up data will be blind to treatment status. In addition there will be specific instructions to participants and clinical teams not to disclose treatment details details at any stage throughout the study. Every effort will be made to maximise the single blindness of research workers, whose 'best guesses' of service user status will be assessed after the final assessment/at the end of the study to test whether their blindness has been maintained. Secondly, there may be selective dropout in one arm of the study; as such, we will examine whether this occurred in the data analysis. Finally it is not designed as a definitive trial but one which will give broad indications of effectiveness and aid the design of a future trial.

## Abbreviations

AD-SUS: Adult Service Use Scale; AUDIT: Alcohol Use Disorders Identification Test; BPD: Borderline Personality Disorder; CMHT: Community Mental Health Team; EQ-5D: EuroQol 5 Dimensions (quality of life measure); HADS: Hospital Anxiety and Depression Scale; JCP: Joint Crisis Plan; SCIDD-II: Structured Clinical Interview for DSM-IV (v. 2.0); WAI: Working Alliance Inventory; WSAS: Work and Social Adjustment Scale.

## Competing interests

The authors declare that they have no competing interests.

## Authors' contributions

PM obtained funding for the study. All authors contributed to the design of the study. All authors read and approved the final manuscript.
